# Epilation for Trachomatous Trichiasis and the Risk of Corneal Opacification

**DOI:** 10.1016/j.ophtha.2011.06.045

**Published:** 2012-01

**Authors:** Saul N. Rajak, Esmael Habtamu, Helen A. Weiss, Amir Bedri, Teshome Gebre, Asrat Genet, Peng T. Khaw, Robin L. Bailey, David C.W. Mabey, Clare E. Gilbert, Paul M. Emerson, Matthew J. Burton

**Affiliations:** 1 The London School of Hygiene and Tropical Medicine, London, United Kingdom; 2 The Carter Center, Atlanta, Georgia; 3 Light For The World, Vienna, Austria; 4 The Amhara Regional Health Bureau, Bahir Dar, Ethiopia; 5 NIHR Biomedical Research Centre, Moorfields Eye Hospital and UCL Institute of Ophthalmology and UCL Partners AHSC, London, United Kingdom

## Abstract

**Purpose:**

Eight million people have trachomatous trichiasis (TT). The World Health Organization (WHO) recommends entropion surgery for TT regardless of severity. However, epilation is widely practiced for treating minor TT (1–5 lashes touching the globe). We report the frequency and effectiveness of patient-initiated epilation and its relationship to corneal opacity.

**Design:**

Cross-sectional baseline data of individuals recruited to 2 randomized, clinical trials.

**Participants:**

We included 2556 individuals (4310 eyes) with previously unoperated TT in ≥1 eye.

**Methods:**

A single ophthalmologist examined all participants for signs of trachoma using WHO grading systems with additional assessment of entropion grading, location and number of trichiatic lashes, and evidence of epilation. A questionnaire enquired about epilation practices.

**Main Outcome Measures:**

The association between epilation and degree of corneal opacity. Epilation practices of TT patients.

**Results:**

Central corneal scarring was present in 1436 (33%) eyes. Entropion was absent/mild in 2328 (54%) eyes, moderate in 1259 (29.2%), and severe in 723 (16.8%). The median number of lashes touching the eye was 2 (interquartile range, 1–5; range, 0–133). There was clinical evidence of epilation in 3018 (70%) eyes, of which 738 (24%) were successfully epilated (no lashes touching globe). Epilation was performed frequently (at least monthly in 3311 [76.8%] eyes), by someone other than the patient (92.8%), and using locally made forceps (88.9%). Controlling for age and degree of entropion, successful epilation was associated with less corneal opacity (odds ratio [OR], 0.61; 95% confidence interval [CI]. 0.43–0.88; *P* = 0.007). The association was only significant in patients with severe entropion (OR, 0.07; 95% CI, 0.02–0.25; *P*<0.005).

**Conclusions:**

We found an association between successful epilation and less central corneal opacity. This indicates the importance of preventing eyelashes from touching the cornea, particularly in individuals with severe entropion. This is a cross-sectional study; therefore, a causative relationship cannot be concluded. However, the results suggest that among patients who decline or are unable to access surgery, and perhaps in minor TT where the management remains controversial, the provision of high-quality forceps and epilation training may be beneficial.

**Financial Disclosure(s):**

The authors have no proprietary or commercial interest in any of the materials discussed in this article.

Worldwide, there are estimated to be over 40 million people with active trachoma and a further 8 million with trachomatous trichiasis (TT).^1^ Active trachoma is characterized by follicular and papillary inflammation of the upper tarsal conjunctiva, caused by *Chlamydia trachomatis*. This recurrent, chronic inflammation leads to conjunctival scarring, which results in cicatricial entropion and trichiasis (lashes touching the eye). Repeated abrasion and corneal ulceration causes corneal opacification and vision loss. This is effectively irreversible; corneal transplant services are rarely available in trachoma endemic settings, and there is a moderate-to-high risk of graft rejection in trachoma patients, owing to corneal vascularization.^2^ More than 2 million people are thought to be blind or severely visually impaired from trachoma.^3^

The World Health Organization (WHO) recommends entropion surgery for all TT patients, regardless of severity.^4^ The rationale for this is that mild trichiasis may worsen over time and individuals may not re-present for surgery when the problem is more severe. Therefore, preemptive surgery may prevent future blindness. However, surgery is not without problems. Although low trichiasis recurrence rates can be achieved in highly supervised surgical trials, outcomes are probably much worse under operational conditions, where recurrence rates of up to 60% have been reported.^5–12^ Moreover, operative uptake rates are often low, perhaps in part because of poor operative outcomes and complications.^13–19^ As a result, the optimal management of trichiasis, particularly mild disease, remains uncertain and controversial.

As many as 50% of patients with TT have only mild entropion.^12^ The trichiasis may often be because of metaplastic or misdirected eyelashes. Eyelash epilation, which is usually performed with locally made forceps by the patient or a helper, is widely practiced in the community. Some national eye care programs recommend epilation as the primary treatment for minor TT (<6 lashes; e.g., Gambian National Eye Care Program).^20^ Epilation is well tolerated, acceptable to patients, does not preclude subsequent surgery, and is simple to teach. However, epilation needs to be carried out repeatedly, does not correct underlying entropion; it has been suggested that epilation may leave short, broken lashes that might be more damaging than the original long, mobile lashes and thereby worsen corneal opacity.^21,22^

Given the huge burden of untreated trichiasis and the shortcomings and poor uptake of surgery, alternative treatment approaches, particularly for mild disease, would be helpful.^1^ One recent cross-sectional study provided the first indication that epilation may be protective against corneal opacification from trichiasis; in patients with moderate or severe entropion, epilation was associated with a lower risk of corneal opacification.^23^ Before performing a formal trial of epilation as an alternative management strategy to surgery for minor trichiasis, we sought further evidence for a modifying effect of epilation on corneal opacity by assessing the epilation practices of patients and the clinical appearance of their corneas.

## Methods

This study was approved by the National Health Research Ethics Review Committee of the Ethiopian Ministry of Science and Technology, the London School of Hygiene and Tropical Medicine Ethics Committee, and the Emory University Institutional Review Board. Informed consent for the study was taken at the time of enrolment. The research adhered to the tenets of The Declaration of Helsinki.

Individuals with previously unoperated trichiasis were identified through a series of 17 surgical outreach campaigns in 6 districts (*woredas*) in Amhara National Regional State, Ethiopia. The campaigns were advertised in local markets, churches, and schools. Additionally, health extension workers, who are present in every subdistrict (*kebele)* in Ethiopia, were trained to recognize trichiasis and visited homes and villages to look for patients. All eligible patients with unoperated TT were recruited. Individuals were excluded from this study if they were pregnant (self-reported), the trichiasis had been previously operated (was recurrent), or the person was <18 years old.

### Clinical Assessment

A field worker administered a questionnaire in Amharic. Height and weight were measured. Unaided (no patients had spectacles for distance correction) and pinhole logarithm of the minimal angle of resolution visual acuities were measured at 4 m, using an Early Treatment of Diabetic Retinopathy Study equivalent Tumbling E logarithm of the minimal angle of resolution chart (Hong Kong Low Vision Centre).^24^ Examinations were all conducted in a darkened room by a single ophthalmologist (S.N.R.) using 2.5× magnification loupes and a bright torch. Signs of trachoma were graded using an augmented version of the Detailed WHO Trachoma Grading System (follicles, papillae, cicatricae [FPC]).^21^ In addition to the standard FPC grading, the following features were documented: Number of trichiatic lashes and place where the lashes touched the eye in the primary position of gaze (cornea, lateral conjunctiva, and medial conjunctiva). The total number of lashes touching the eye (“lash burden”) was calculated by summing the number touching the medial and lateral bulbar conjunctiva and the cornea. Clinical evidence of epilation was identified by the presence of broken or newly growing lashes, or areas of absent lashes. Upper lid entropion was graded by assessing the degree of inward rotation of the eyelid margin, as described in Fig 1 (available at http://aaojournal.org). The plica semilunaris was examined for scarring or effacement. The presence of symblepharon was recorded. Central corneal opacity was defined as opacity within the central 4 mm of the cornea (grade CO-2a or greater). Corneal scarring was graded in detail (Fig 1). The eyelid was everted and the location of the mucocutaneous junction recorded. The mucocutaneous junction is the junction between keratinized skin epithelium and nonkeratinized conjunctival epithelium. This normally lies posterior to the meibomian gland orifices in the normal lid. Tarsal conjunctival papillary inflammation, follicles, and scarring were graded using the WHO FPC system. The presence of lagophthalmos was assessed by asking the patient to gently close the eyes. All subjects underwent treatment for their trichiasis as part of ongoing clinical trials, on the same day as their examination.

### Data Analysis

Data were double entered into an Microsoft Access database and analyzed using Stata 10 (SataCorp, College Station, TX). Cuzick's nonparametric test was used to test for trend across ordered groups. Generalized linear latent and mixed models (GLLAMM) were used to adjust for within-person correlation by fitting random-intercept models. Logistic regression GLLAMM models were used to assess the association of binary outcomes with exposures, and ordinal logistic regression GLLAMM models were used to assess ordered categorical outcomes (corneal opacity grade) with exposures. For the latter variables which were associated with the outcome on univariate analyses (*P*<0.2) were retained in the multivariate model. Entropion grade, which is not thought to be affected by epilation, was used as a marker of disease severity because lash burden (number of lashes touching the globe) is altered by epilation. It is, therefore, inappropriate for stratification when comparing outcomes in those who do and do not epilate. For the logistic regression models, age was divided into the following categories: 18 to 19, 20 to 29, 30 to 39, 40 to 49, 50 to 59, 60 to 69, and >70 years. In models that examine the association between epilation and corneal opacity, the results of the clinical examination for epilation are used, rather than the clinical history of epilation.

## Results

We recruited 2556 consecutive patients presenting with previously unoperated trichiasis in ≥1 eye. No patient refused participation in the study. Among these patients, there were 4310 eyes with previously unoperated trichiasis (74 of the patients had had previous surgery in 1 eye, but the second eye had unoperated TT and was used in this analysis). Eight hundred six participants had unilateral TT and 1752 had bilateral disease. Right (n = 2151, 49.9%) and left eyes (n = 2159, 50.1%) were equally affected. The distribution of number of lashes touching the eye, severity of entropion and the corneal scar grade are presented in Table 1. Central corneal opacity was found in 404 of 723 participants (55.9%) with severe entropion. In patients with no or mild entropion, central corneal opacity was found in 569 of 2328 participants (24.4%). The median age was 50 (interquartile range, 40–60) and 1845 (72%) were women. The vast majority of participants (2555/2556) self-identified as ethnic Amharan Ethiopian. Most (2368, 93%) were illiterate (98% of women and 77% of men). The median body mass index was 19.9 kg/m^2^ (interquartile range, 18.4–21.4; total range, 12.5–33.7).

Epilation practice was ascertained from the history and examination. Interview responses are shown in Table 2 and the clinical examination findings in Table 3. There was strong agreement between the number of patients thought to epilate by clinical examination and by patient history (*P*<0.0001; Table 4). The agreement is greatest for those who report epilating within the last week and least for those who report epilating more than a month before. However, there was some disparity: Among those who denied epilation, there was clinical evidence of epilation in 176 of 872 eyelids (20.2%) and in participants who did report epilation, there was no clinical evidence of it having had occurred, in 597 of 3438 eyelids (17.4%).

Individuals were more likely to epilate if they have severe entropion (*P*<0.0001). The associations between central corneal opacity (grade ≥2) and any epilation and between central corneal opacity and successful epilation (in eyes with evidence of any epilation) are presented in Table 5. The rate of central corneal opacity is almost identical in patients who do and do not epilate. However, the rate of central corneal opacity is lower in those who epilate successfully (24.4%) compared with those who epilate but have some residual lashes (36.1%). Multivariable ordinal logistic regression modeling (Table 6) indicates that successful epilation is associated with less corneal opacity, although increasing entropion severity and age are associated with increasing severity of corneal opacity. The association between epilation and corneal opacity was investigated for 3 entropion severity states (none [E0]/mild [E1], moderate [E2], or severe [E3/E4]). Unsuccessful epilation and successful epilation are both associated with less corneal opacity in more severe entropion disease states and there is a nonsignificant association between successful epilation and decreased corneal opacity in less severe disease states (Table 7).

## Discussion

Self-initiated epilation is widely practiced in trachoma endemic areas. Seventy percent of patients in the present study had clinical evidence of epilation on examination. Similarly high rates (69%–82%) have been reported from Ethiopia and elsewhere.^25–27^ Successful epilation (all trichiatic lashes removed) is strongly associated with less corneal opacity, whereas incomplete epilation shows a nonsignificant trend toward less opacity. This indicates the importance of preventing eyelashes from touching the cornea. In patients with severe entropion, both successful and incomplete epilation were strongly associated with less corneal opacity. The difference in the strength of the association between epilation practice and an apparent reduction in the risk of corneal opacification for different degrees of entropion, may be attributable to the lower frequency of central corneal opacification in the mild group.

A similar relationship was found in a cross-sectional study of epilation and corneal opacity from Ethiopia by West et al.^23^ They report a significant association between epilation and opacity in moderate (34.4% corneal opacity in nonepilators versus 21.3% in epilators; *P* = 0.002) and severe disease (74% vs 43%; *P* = 0.0001) but not in mild disease (8.9% in nonepilators versus 10.9% in epilators; *P* = 0.23).

It has been said that poorly executed epilation can leave sharp, broken lashes that may be more damaging to the cornea than the original long, “soft” lashes.^21,22^ We did not find evidence to support this view and in patients with severe entropion, even unsuccessful epilation was associated with less corneal opacification. Epilating whole lashes and their roots completely from their follicles is preferable, because this extends the time before they return.

Trachomatous trichiasis causes visual loss through corneal opacification, probably by direct lash trauma and secondary bacterial or fungal infection. Trachomatous trichiasis can also cause severe pain, photophobia, and epiphora. In many trachoma endemic settings, there is a chronic shortage of TT surgical services. For example, in 2009 the estimated backlog of individuals with TT in the Amhara region of Ethiopia where the present study was conducted was over 490 000. In 2009 and 2010, only 72 000 received TT surgery. Even when surgery is available, it is not without problems. Surgical recurrence rates can be as high as 62% in operational settings and minor complications such as granuloma and poor cosmetic outcome occur relatively frequently.^8,28^ Perhaps in part because of uncertain surgical outcomes, even in settings where free surgery is available, surgical uptake is often low.^13–19^

The optimal management of minor TT (<6 trichiatic lashes) is controversial. The WHO advises lid rotation surgery for all TT patients irrespective of severity, even if only 1 peripheral lash touches the eye.^4^ However, many ophthalmologists and some trachoma control programs advise epilation for minor trichiasis. There are no reported trials comparing surgery and epilation, and a randomized, controlled trial addressing this question would be useful. Although the present study did not demonstrate an association between reduced corneal opacity and epilation in patients with mild or no entropion, the rates of central corneal opacity were relatively small in this group. Therefore, because individuals with few lashes often decline surgery, epilation may be prudent until the disease worsens.

The regularity with which participants in our study performed epilation (at least weekly by 41%) suggests that it is well tolerated and that it alleviates some of the symptoms of pain, epiphora, and photophobia that accompany trichiasis. However, the high rate of incomplete epilation indicates that it is either poorly executed or not performed sufficiently frequently. Furthermore, in contrast with resource-rich countries where epilation of trichiatic lashes is routinely carried out in ophthalmic clinics, no patient in this study had their epilation performed by a health care professional. Epilation was mainly conducted with locally made forceps. These are usually fashioned from an old piece of metal, often do not oppose squarely, and have sharp or jagged edges and tips. Additionally, patients or their helper receive no training in epilation. The poor quality of the available epilating forceps probably contributes to the low proportion of lids that were “successfully” epilated. If, as this study suggests, successful epilation can reduce the risk of corneal opacification, the outcome may be improved by the use of high-quality, round-edged forceps with opposing tips and the training of a well-sighted helper.

In the absence of directly observed trichiasis, the decision on whether to perform lid rotation surgery is often guided by the history and signs of epilation. However, these are not always concordant. A disparity between the patient's reported history of epilation and clinical evidence of epilation can occur either because the evidence of epilation is absent or missed on clinical examination, or because the patient misreports epilating. If the epilation occurred a long time previously and all lashes have grown back to a normal length or the patient epilated very recently and just a few lashes were removed from their follicles without breakage, epilation is not apparent on examination. Conversely patients may falsely report epilation in a misguided attempt to receive surgical treatment. Therefore, a clear history and careful examination are needed to determine whether surgery is appropriate.

This is a cross-sectional study and as such it has limited capacity to elucidate the relationship between corneal opacity and epilation. In examining the association between epilation and the risk of central corneal opacity, disease severity is a confounding factor. Patients with more severe trichiasis are more likely to epilate and at the same time are also more likely to have central corneal opacity. Trichiasis burden (number of lashes) is the most widely used measure of disease severity. However, epilation alters the number of lashes. Therefore, the number of lashes is not a reliable measure of the severity of disease. To try to overcome this limitation, we have used entropion severity as an alternative measure of disease severity for stratification in logistic regression models. Despite stratifying by entropion severity, confounding may still occur within each stratification group. For example, among patients with entropion grade 1, it is possible that those at the milder end of the grade 1 spectrum are less likely to epilate than those at the more severe end. Therefore, the actual risk reduction effect of epilation on central corneal opacity may be underestimated. Clinical examination of epilation provides a “snapshot” of current epilation practice. It is possible that corneal opacity formed during periods of poor or infrequent epilation. If this were the case, the association between epilation and reduction in corneal opacity would be stronger. A long-term, prospective study would be required to investigate this.

Lid rotation surgery is the best available treatment for moderate or severe trichiasis, especially when there is associated entropion. The optimal management of mild trichiasis is less certain. The association between epilation and a lower risk of corneal opacity suggests that epilation might protect the cornea of patients with trichiasis. Further studies are required to formally investigate the optimal management of mild trichiasis. However, for the many patients who decline surgery or who are unable to access surgery, the provision of high-quality forceps and epilation training is likely to be of benefit.

## Figures and Tables

**Table 1:** Clinical Features: Number of Lashes Touching Eye (Lash Burden), Entropion Grade, and Corneal Scarring in 4311 Eyes with Previously Unoperated Trichiasis

Clinical Feature	Number	%
Lash burden		
Median	2	
Range	0–133	
Interquartile range	1–5	
0 (successful epilation)	738	17.1
1–5	2495	57.9
6–9	542	12.6
10–19	335	7.8
≥20	200	4.6
Entropion grade		
0	1016	23.6
1 (mild)	1312	30.4
2 (moderate)	1259	29.2
3 (severe)	416	9.7
4 (severe)	307	7.1
Corneal scar grade		
0	1675	38.9
1	1199	27.8
2a	719	16.7
2b	132	3.1
2c	345	8.0
2d	97	2.2
3	117	2.7
Phthisis	26	0.6

**Table 2:** History of Epilation at Presentation in 4311 Eyes Considered to Have Previously Unoperated Trichiasis

Question and Response Options	N	%
On average, how frequently is epilation performed?		
Never	872	20.2
Less than once/month	127	3.0
Between once/week and once/month	1549	35.9
Once/week or more	1762	40.9
When was epilation last performed?		
Never	872	20.2
>30 days ago	370	8.6
7–30 days ago	1584	36.8
Within last 7 days	1484	34.4
Who performs the epilation?		
No history of epilation	872	20.2
The patient	248	5.8
A friend, relative, or neighbor	3190	74.0
Method of epilation		
No history of epilation	872	20.2
Locally handmade forceps	3057	70.9
Machine made forceps	38	0.9
Burning with hot coals	178	4.1
Cutting	6	0.2
Pulling with fingers	159	3.7

**Table 3:** Clinical Evidence of Epilation on Examination

Characteristic	Number of Eyes	%
Epilation		
No epilation	1293	30.0
<One third of lashes	2087	48.4
One to two thirds of lashes	438	10.2
>Two thirds of lashes	492	11.4
Epilation success⁎		
No clinical evidence of epilation	1293	30.0
No lashes touching eye (successful)	738	17.1
<6 lashes touching eye	1600	37.1
≥6 lashes touching eye	679	15.8

⁎ Based on clinical determination of epilation, and not patient's history of epilation.

**Table 4:** Relationship between Clinical Evidence of Epilation on Examination and Last Reported Episode of Epilation

Clinical Evidence of Epilation	Patient's Report of Epilation				
	Not Epilating	Epilated >30 Days Ago	Epilated 7–30 Days Ago	Epilated within Last 7 Days	Total
Yes	176 (20.2%)	165 (44.6%)	1279 (80.7%)	1397 (94.4%)	3017 (100%)
No	696 (79.8%)	205 (55.4%)	305 (19.3%)	87 (5.9%)	1293 (100%)
Total	872	370	1584	1484	4310

**Table 5:** Relationship Between Any Epilation and Corneal Opacity and Successful Epilation and Corneal Opacity

Corneal Opacity Grade	Eyes with Clinical Evidence of Epilation⁎		Eyes with Successful Epilation†‡	
	Yes	No	Yes	No
0, 1	2010 (66.7%)	863 (66.5%)	552 (75.6%)	1460 (63.9%)
≥2	1003 (33.3%)	435 (33.5%)	178 (24.4%)	826 (36.1%)

⁎ Total number of eyelids = 4311.

† Successful epilation is defined as clinical evidence of epilation and no lashes touching the globe.

‡ Patients who do not epilate excluded. The denominator is 3016 lids with clinical evidence of epilation.

**Table 6:** Univariate and Multivariate Generalized Linear Latent and Mixed Model Logistic Regression Models for Central Corneal Opacity⁎

Variable	Pupil Obscuring/Central Corneal Opacity		
	OR^§^	95% CI	P Value
Univariate analysis			
Gender			
Male	1	—	—
Female	0.81	0.61–1.08	0.154
Advancing age†	1.52	1.39–1.67	<0.005
Epilation status			
No epilation	1	—	—
Unsuccessful epilation	1.18	0.92–1.52	0.200
Successful epilation	0.57	0.40–0.81	0.002
Entropion grade‡			
0	1	—	—
1	1.54	1.12–2.12	0.008
2	3.55	2.54–4.96	<0.005
3	8.36	5.34–13.09	<0.005
4	44.98	26.20–77.20	<0.005
Multivariate logistic regression model			
Advancing age	1.63	1.48–1.80	<0.005
Unsuccessful epilation	0.87	0.66–1.13	0.29
Successful epilation	0.61	0.43–0.88	0.007
Entropion grade‡			
0	1	—	—
1	1.68	1.22–2.31	0.002
2	4.15	2.93–5.86	<0.005
3	9.49	6.05–14.86	<0.005
4	45.07	26.29–77.26	<0.005

CI = confidence interval; OR = odds ratio.

⁎ For the generalized linear latent and mixed model outcome, corneal opacity subdivided into 4 groups: (1) CO 0/1 (no or peripheral opacity), (2) CO 2a/2b (central 4 mm, not overlying visual axis), (3) CO 2c/2d (overlying visual axis, but not obscuring all central 4 mm), and (4) CO 3 (overlying whole central 4 mm). Phthisis (CO 4) excluded.

† Advancing age group has a linear association with increasing corneal opacity.

‡ Entropion grades 1–4 are compared with entropion grade 0.

**Table 7:** Multivariate Logistic Regression Models (Generalized Linear Latent and Mixed Models) for Increasing Corneal Opacity⁎ in Relation to Epilation Status (No, Unsuccessful, and Successful) for the 3 Different Entropion Severity States, Adjusted for Age

	None/Mild Entropion (E0, 1)			Moderate Entropion (E2)			Severe Entropion (E3, 4)		
	OR	95% CI	P	OR	95% CI	P	OR	95% CI	P
No epilation	1	—	—	1	—	—	1	—	—
Unsuccessful epilation	1.39	0.97–2.00	0.074	0.73	0.44–1.22	0.23	0.26	0.14–0.50	<0.005
Successful epilation	0.76	0.47–1.20	0.24	0.80	0.41–1.55	0.51	0.07	0.02–0.25	<0.005

CI = confidence interval; OR = odds ratio.

⁎ For the generalized linear latent and mixed models model outcome, corneal opacity subdivided into 4 groups: (1) CO 0/1 (no or peripheral opacity), (2) CO 2a/2b (central 4 mm, not overlying visual axis), (3) CO 2c/2d (overlying visual axis, but not obscuring all central 4 mm), and (4) CO 3 (overlying whole central 4 mm). Phthisis (CO 4) excluded.
