# Supplementary material for: Epilation for Trachomatous Trichiasis and the Risk of Corneal Opacification
Source: Ophthalmology. 2012 Jan;119(1):84–9. doi: 10.1016/j.ophtha.2011.06.045 (PMC3694301; doi:10.1016/j.ophtha.2011.06.045)
Supplement: Figure 1 [file mmc1.pdf]

Sign		Definition		World Health Organisation (WHO) equivalent grade
Entropion (assessed with eye in primary position)				
E0	(none)	None		
E1	(mild)	<50 % of lid margin rolled inwards without cornea - lash base contact		
E2	(moderate)	>50% of lid margin rolled inwards without cornea –lash base contact		No WHO grading system for entropion
E3	(severe)	Lid margin rolled inwards with <50% lash base – cornea contact		
E4	(severe)	Lid margin rolled inwards with >50% lash base – cornea contact		
Corneal scarring (assessed with eye in primary position)				
CO-0		None		CC0
CO-1	Opacity Location:	Opacity, outside central 4mm of cornea	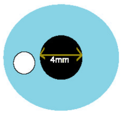	CC1
	Opacity Density:	Any		
CO-2a	Location:	Within central 4mm, but not entering central 1mm.	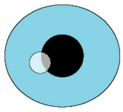	
	Density:	Pupil margin visible through opacity.		
CO-2b	Location:	Within central 4mm, but not entering central 1mm.	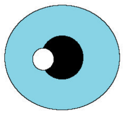	
	Density:	Pupil margin not visible through opacity.		CC2
CO-2c	Location:	Overlying central 1mm, i.e. visual axis	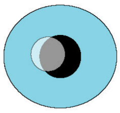	
	Density:	Pupil margin visible through opacity.		
CO-2d	Location:	Overlying central 1mm, i.e. visual axis	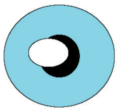	
	Density:	Pupil margin not visible through opacity.		
C0-3	Location:	All of central 4mm	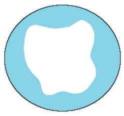	CC3
	Density:	Pupil margin not visible through opacity		
CO-4		Phthisis		No WHO grade

Fig 1: Expanded grading system and WHO grading system equivalents
